# Associations between cardiometabolic traits and diabetic cardiomyopathy: an imaging-based analysis

**DOI:** 10.1007/s00592-026-02687-4

**Published:** 2026-04-15

**Authors:** Georgios Mavraganis, Dimitrios Bampatsias, Christina Konstantaki, Kamil Stankowski, Stavros Athanasopoulos, Chrysoula Moustou, Alexandros Alexandropoulos, Stefano Figliozzi, Angelos Soranides, Ioannis Petropoulos, Dimitrios Klettas, Kimon Stamatelopoulos, Georgios Georgiopoulos

**Affiliations:** 1 Department of Clinical Therapeutics, Alexandra Hospital, National and Kapodistrian University of Athens Medical School, 80 Vas. Sofias Str, Athens, 11528 Greece; 2 Cardiac Amyloidosis Program, Division of Cardiology, Department of Medicine, Columbia University College of Physicians and Surgeons, New York, NY USA; 3 IRCCS Humanitas Research Hospital, Milan, Rozzano Italy; 4 Department of Biomedical Sciences, Humanitas University, Pieve Emanuele, Milan, Italy; 5 First Department of Cardiology, School of Medicine, National and Kapodistrian University of Athens, Hippokration General Hospital, Athens, 11527 Greece; 6 Faculty of Medical Sciences, Biosciences Institute, Vascular Biology and Medicine Theme, Newcastle University, Newcastle Upon Tyne, UK; 7 Second Department of Cardiology, “Attikon” Hospital, National and Kapodistrian University of Athens Medical School, Athens, Greece

**Keywords:** Diabetic cardiomyopathy, Cardiac magnetic resonance, Echocardiography, Arterial stiffness, Ventricular-arterial coupling, Liver fibrosis

## Abstract

**Introduction:**

Diabetic cardiomyopathy (DCM) often evades diagnosis before manifestation of clinical symptoms. In this study we explored how cardiometabolic traits influence early cardiac structure and function in asymptomatic people living with diabetes (PwD), using advanced imaging.

**Methods:**

We conducted a cross-sectional study of 88 participants: 57 people living with type 2 diabetes (PwT2D), 16 people living with type 1 diabetes (PwT1D) and 15 controls. All subjects underwent transthoracic echocardiography and/or cardiac magnetic resonance (CMR) imaging. Strain analysis, perfusion indices, and tissue characterization (T1, T2, and extracellular volume) were assessed. Arterial stiffness via pulse wave velocity (PWV), ventricular-arterial coupling (VAC), circulating biomarkers and liver fibrosis indices were evaluated.

**Results:**

PwD had lower cardiac index than controls. Global longitudinal strain (GLS) and global radial strain were lower in both diabetes mellitus (DM) groups, while left atrial strain was most impaired in PwT2D (β-coefficient= − 11.77, *P*= 0.003). DM duration ≥ 10 years was associated with worse GLS (β-coefficient= − 2.18, *P*= 0.033) and right VAC (β-coefficient= − 0.27, *P*= 0.027) after multivariable analysis. While tissue characterization and perfusion indices showed no significant group differences, tight glycemic control in PwD correlated with improved myocardial strain parameters. PwT2D exhibited greater arterial stiffness (β-coefficient= 1.52, *P*= 0.003). In PwD, elevated non-alcoholic fatty liver disease score correlated with increased left ventricular mass (β-coefficient= 6,195, *P*= 0.022) and decreased left ventricular ejection fraction (LVEF) (β-coefficient= − 3.12, *P*= 0.017). Higher growth differentiation factor levels were associated with reduced LVEF (β-coefficient= − 0.005, *P*= 0.029).

**Conclusion:**

This multimodal imaging study highlights myocardial and vascular changes in asymptomatic PwD. Early comprehensive cardiovascular assessment may help identify dysfunction before overt heart failure develops.

**Supplementary Information:**

The online version contains supplementary material available at 10.1007/s00592-026-02687-4.

## Introduction

Diabetic cardiomyopathy (DCM) is a clinical condition characterized by structural and functional alterations of the myocardium in people living with diabetes (PwD), occurring independently of the presence of coronary artery disease, hypertension, or other common causes of cardiomyopathy [1]. DCM is now recognized as a significant contributor to the increased burden of heart failure (HF), particularly HF with preserved ejection fraction (HFpEF) in people living with type 2 diabetes (PwT2D) [2]. Given the steadily growing prevalence of type 1 diabetes mellitus (T1DM) and type 2 diabetes mellitus (T2DM) globally, understanding the pathophysiological mechanisms and enabling early detection of DCM has become increasingly important given also the differential underlying pathophysiology in T1DM and T2DM [3–5].

Early identification of subclinical cardiovascular (CV) dysfunction in PwD is critical, as structural and functional abnormalities may precede overt clinical cardiovascular disease (CVD) by several years. In this context, validated non-invasive surrogate imaging biomarkers such as global longitudinal strain (GLS) and pulse wave velocity (PWV) provide sensitive measures of early myocardial dysfunction and vascular remodeling [6, 7]. These parameters have been increasingly recognized as early markers of DCM and may facilitate earlier risk stratification and targeted intervention in asymptomatic high-risk populations [8, 9]. Importantly, the use of validated surrogate imaging endpoints may also facilitate earlier CV risk stratification and support therapeutic targeting in high-risk populations such as PwD, where CV damage may develop despite absence of symptoms.

The pathogenesis of DCM is multifactorial, driven by interactions among metabolic, inflammatory, and neurohormonal mechanisms. Key cardiometabolic factors-including insulin resistance, dyslipidemia, central obesity, elevated blood pressure, and persistent low-grade inflammation- play a crucial role in the myocardial impairment observed in DCM [10, 11]. These factors promote processes such as myocardial fibrosis, lipotoxicity, disrupted energy metabolism, and abnormal calcium handling, which can occur before the onset of clinically evident systolic or diastolic dysfunction [2].

Traditional diagnostic tools often fail to detect early myocardial changes in asymptomatic individuals, highlighting the need for more advanced imaging modalities which can capture such alterations [12]. Advances in CV imaging -particularly cardiac magnetic resonance (CMR) imaging and echocardiographic strain imaging- have significantly improved the ability to characterize myocardial structure, function, and tissue composition non-invasively [13]. CMR, with its superior spatial resolution and tissue characterization capabilities, is regarded as the gold standard for evaluating cardiac morphology and fibrosis [14]. Techniques such as T1 mapping, extracellular volume (ECV) quantification, and late gadolinium enhancement (LGE) have allowed the detection of possible subclinical myocardial remodeling, even in the absence of symptoms or functional decline [15]. In the context of early CV remodeling, arterial stiffness measured by pulse wave velocity (PWV) has also shown a consistent association with HFpEF [16].

Despite the utility of these imaging modalities, differential imaging markers may capture different stages and phenotypes of DCM [17]. Previous studies have investigated the effects of individual metabolic markers on cardiac structure and function; however, there is a lack of comprehensive evaluations that combine multiple cardiometabolic traits with advanced imaging parameters [18]. Delineating these associations has the potential to advance our understanding of the biological and clinical heterogeneity of DCM, refine risk stratification, and ultimately inform the rational design and allocation of targeted interventions for individuals at heightened risk of HF.

Beyond the advances in imaging that may enable earlier detection of preclinical DCM, DM itself is now increasingly recognized as a heterogeneous condition. DM often overlaps with metabolic dysfunction-associated steatotic liver disease and an association between liver fibrosis non-invasive test (LFNITs) and atherosclerotic CV disease has been demonstrated [19, 20]. Recent efforts to disentangle its diverse pathophysiological mechanisms have underscored a common clinical denominator -disease duration- together with overlapping pathways that converge on CV dysfunction [2]. These insights not only refine our understanding of risk trajectories but also underscore the importance of selecting antidiabetic therapies with demonstrated cardioprotective benefits [21] Importantly, longer DM duration has been linked to progressive myocardial remodeling, characterized by greater degrees of myocardial fibrosis and diastolic dysfunction, as assessed by advanced imaging modalities including cardiac T1 mapping and echocardiographic strain imaging [22] Furthermore, sodium-glucose co-transporter 2 inhibitors (SGLT2i) have shown promising cardioprotective effects in PwD, potentially attenuating myocardial fibrosis and reducing ECV which can be both visualized through advanced imaging markers like ECV quantification and GLS [23, 24]. These findings highlight the importance of considering both diabetes duration and pharmacologic therapy when evaluating subclinical myocardial changes in DCM.

In this study, we aimed to investigate the associations between a spectrum of cardiometabolic traits, including LFNITs, and imaging-defined markers of DCM using advanced CV imaging, i.e. comprehensive transthoracic echocardiography and CMR imaging.

## Methods

### Population

**Fig. 1:**
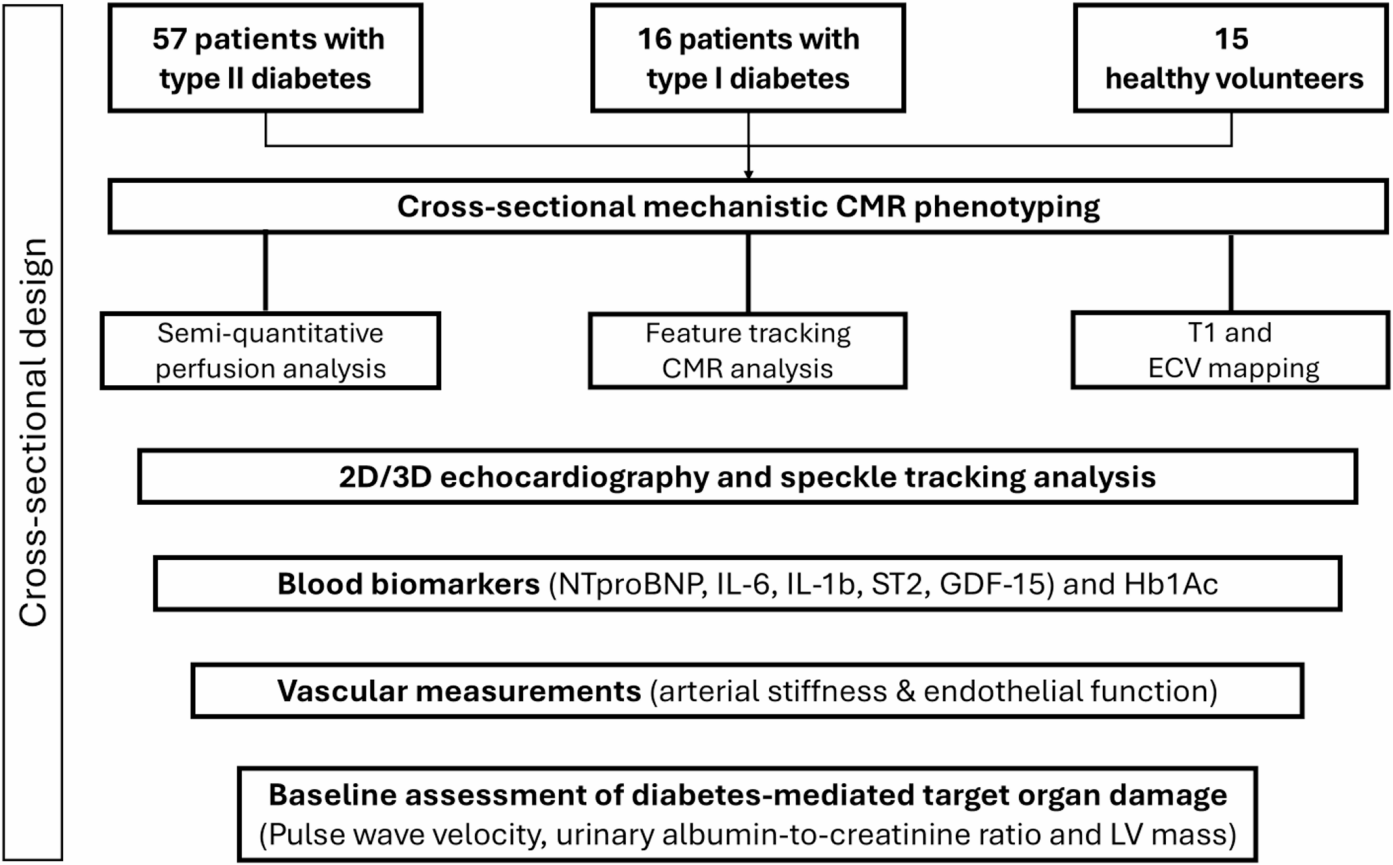
Overview of study design. Abbreviations: *CMR* cardiac magnetic resonance, *ECV* extracellular volume, *NT-proBNP* N-terminal pro–B-type natriuretic peptide, *IL-6* interleukin-6, *IL-1b* interleukin-1b, *GDF-15* growth differentiation factor-15, *Hb1AC* hemoglobin A1c, *LV* left ventricular

This is a single-center cross-sectional study that included 57 PwT2D, 16 PwT1D and 15 controls without presence of DM and clinically overt CV disease who underwent echocardiographic assessment and/or CMR imaging between January 2022 and December 2023 at the Department of Clinical Therapeutics of National and Kapodistrian University of Athens (Alexandra General Hospital). Participants were recruited consecutively among those attending the clinic who met inclusion criteria. Figure 1 depicts the study design.

**Fig. 2:**
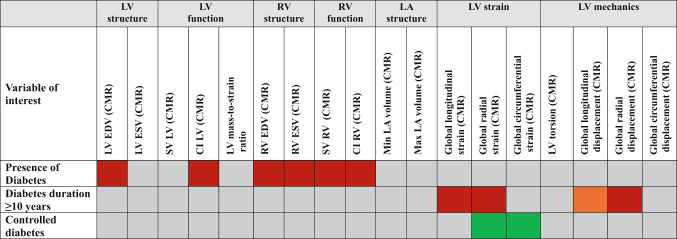
Associations of diabetes characteristics with structural and functional markers of cardiac function. Abbreviations: *CMR* cardiac magnetic resonance, *LV* left ventricular, *EDV* end-diastolic volume, *ESV* end-systolic volume, *SV* stroke volume, *CI* cardiac index, *RV* right ventricular, *LA* left atrium. Red bars correspond to inverse associations after multivariable adjustment, orange bar corresponds to inverse association at univariable analysis and green bars correspond to positive association after multivariable adjustment

All participants provided written consent for participation in the study. The current study was conducted according to the principles of the Declaration of Helsinki and the Local Ethics Committee of Alexandra General Hospital approved the study’s protocol (19.07.2021/1379).

### Echocardiography

Patients enrolled in the study underwent transthoracic echocardiography, which included both conventional echocardiographic imaging and acquisitions suitable for speckle tracking analysis. All imaging was conducted by a single, highly experienced operator using a commercial echocardiography system (Vivid S70; GE Medical Systems, Milwaukee, WI, USA). The images were obtained in accordance with the recommendations of the European and American Associations of Echocardiography [25]. Details are provided in the Supplementary Material.

### Cardiac magnetic resonance imaging

For patients who underwent a CMR examination, a 3.0T MRI Philips (Achieva TX) scanner was used. CMR analysis was performed by a radiologist experienced in CMR imaging and one magnetic resonance imaging (MRI) physicist blinded to participants’ clinical history and profile using the commercially available software (Circle cmr42 release 5.11.4; Circle Cardiovascular Imaging, Calgary, AB, Canada). CMR acquisition protocol and analysis methods to obtain parameters of LV structure, function and myocardial mapping to derive global T1, ECV and T2 maps are described in detail in previously published manuscripts [26, 27]. A full description for methodology is provided in Supplementary Material.

### Peripheral vascular studies

Arterial stiffness was evaluated by carotid-femoral PWV and ventricular-arterial coupling (VAC) by the PWV to global longitudinal strain (PWV/GLS). A detailed description of the assessment of aortic stiffness has been previously published [28]. More details for VAC are provided in the Supplementary Material.

### Metabolic profile, liver fibrosis and biomarkers measurement

All participants underwent standardized clinical and laboratory assessment, including metabolic profiling and calculation of liver fibrosis indices. Tight glycemic control was defined as hemoglobin A1c ≤ 6.5%. Circulating biomarkers were measured using validated assay platforms. Detailed procedures are provided in the Supplementary Material.

### Intra-observer variability

The reliability of intra-observer measurements for native T1 was evaluated by performing a repeatability analysis of native T1 in a random subset of 20 patients. The same reader, blind to initial results, repeated measurements separated in time from the first analysis. Furthermore, intraobserver variability for PWV measurements was evaluated in another randomly selected subset of 20 participants by repeated offline analysis performed by the same observer, blinded to the original results, using the same acquisition and analysis protocol. Intra-observer repeatability for native T1 was excellent, with an intraclass correlation coefficient (ICC) of 0.93 (95% CI 0.91–0.95). Moreover, coefficient of variation for two repeated measurements of PWV was 2.4%.

### Outcomes

The primary outcomes of this study were GLS assessed by echocardiography and carotid-femoral PWV as a marker of arterial stiffness, which were prespecified and powered for the current sample size. All other parameters —including cardiac structure and function, myocardial mechanics, CMR-derived tissue characterization, arterial-ventricular coupling markers, liver fibrosis scores and blood biomarkers— were considered secondary or exploratory outcomes.

### Statistical analysis

Normality of continuous variables was graphically assessed by histograms and P-P plots. For continuous variables, normally distributed data were expressed as means ± standard deviation (SD). Non-normally distributed data were presented as median and interquartile range. Pairwise differences were evaluated using the independent samples Student’s T test or the Mann-Whitney test for continuous variables and the chi-squared test for categorical ones. To compare variables across more than 2 groups (i.e., PwT2D vs. PwT1D vs. controls), one-way analysis of variance (ANOVA) was employed [29]. For categorical variables, the Chi-Square or Fisher exact test were used to compare the distributions for the two randomized groups.

We employed regression analysis (i.e. linear regression and logistic regression) to evaluate the independent association between DM status and markers of DCM -assessed either by transthoracic echocardiography or CMR imaging-, arterial stiffness and VAC, after adjusting for traditional risk factors (TRFs) including age, sex and hypertension. Multivariable models were built based on biological plausibility. To minimize model overfitting given the sample size, multivariable models were restricted to a prespecified core set of clinically relevant covariates (age, sex, and hypertension) [30]. Analyses were conducted using complete-case datasets, and the number of participants included in each model is reported in the Supplementary Material. Estimated odds ratios (OR) and coefficients with the respective 95% confidence intervals (CI) were documented.

Sample size estimation was performed a priori based on the co-primary study endpoints, namely GLS and PWV. The calculation was informed by previously reported effect sizes for differences in myocardial strain and arterial stiffness between PwD and non-diabetic controls in studies using advanced CV imaging. Based on these assumptions, the target sample size was considered sufficient to detect clinically meaningful differences in GLS and PWV with adequate statistical power. Power calculations were performed based on GLS and PWV, which were prespecified as the co-primary outcomes. Based on the number of participants, the study had 80% power to detect a clinically meaningful difference of ≥ 2% in GLS and 1 m/s difference in PWV, assuming a two-sided alpha level of 0.05. All other outcomes were considered exploratory, and the study was not formally powered to detect differences in these parameters. Measures of dispersion used for the calculation were derived from previously published studies and assumed a SD of approximately 2.5–3.0% for GLS and 1.2–1.5 m/s for PWV in diabetic populations undergoing advanced CV imaging [31–34]. The co-primary study endpoints (GLS and PWV) were pre-specified a priori and analyzed as hypothesis-driven outcomes. Given the exploratory and hypothesis-generating nature of secondary analyses, formal Bonferroni correction (or FDR) for multiple comparisons was not applied. This approach was chosen to reduce the risk of type II error in the context of advanced imaging phenotyping and is consistent with prior exploratory biomarker studies. Therefore, secondary analyses should be interpreted as exploratory. Anticipated differences in variables of interest were evaluated using the non-parametric Wilcoxon Mann Whitney test. Type I error was predefined at 0.05. Power calculations were performed with G*Power 3.1.9.6 (University of Kiel).

All significance tests were two tailed and conducted at the 5% significance level. All statistical analyses were performed with SPSS (version 28.0; SPSS, Chicago, IL) and STATA v18.0 (STATACorp LLC).

## Results

Demographic characteristics of the study’s population are summarized in Table 1. The mean age of PwT2D was 64.1 (± 7.9) years, while PwT1D were significantly younger, with a mean age of 46.2 (± 7.9) years, and controls had a mean age of 54 years (± 15.9). Regarding disease duration, mean time from diabetes diagnosis was 11.2 (± 8.5) years for the T2DM group and 21.7 (± 10.9) years for the T1DM group. Moreover, the prevalence of hypertension was higher in PwT2D compared with PwT1D and controls (54.4% vs. 6.3% vs. 26.7%, *P* < 0.001). CMR and echocardiographic parameters according to DM status are presented in Tables 2 and 3 respectively. Unless otherwise specified, echocardiography-derived and CMR-derived parameters are reported separately throughout the results and corresponding tables to avoid misinterpretation due to modality-specific measurement differences. Regarding GLS, strain values were analyzed and reported as absolute values to facilitate clinical interpretation and comparability across modalities.

**Table 1:** Descriptive characteristics of the study population

Characteristic	Total population (*n* = 88)	Healthy controls (*n* = 15)	Type 1 Diabetes (*n* = 16)	Type 2 Diabetes (*n* = 57)	*P*-value
Age (years)	59.2 (11.9)	54.4 (15.9)	46.2 (7.9)	64.1 (7.9)	< 0.001
Sex (male) (*n*, %)	56 (63.6)	10 (66.7)	10 (62.5)	36 (63.2)	0.964
BMI (kg/m^2^)	28.0 (4.7)	27.9 (6.5)	26.2 (3.6)	28.6 (4.4)	0.213
Smoking (*n*, %)	22 (25.0)	1 (6.7)	5 (31.3)	16 (28.1)	0.191
Diabetes duration (years)	13.3 (10.0)	N/A	21.7 (10.9)	11.2 (8.5)	< 0.001
Hypertension (*n*, %)	36 (40.9)	4 (26.7)	1 (6.3)	31 (54.4)	< 0.001
Hyperlipidemia (*n*, %)	69 (78.4)	8 (53.3)	12 (75)	49 (86.0)	0.022
Alcohol consumption (*n*, %)	17 (19.3)	1 (6.7)	3 (18.8)	13 (22.8)	0.370
Atrial fibrillation (*n*, %)	5 (5.7)	2 (13.3)	0 (0)	3 (5.3)	0.270
Hypolipidemic treatment (*n*, %)	57 (64.8)	5 (33.3)	8 (50)	44 (77.2)	0.004
ACEi/ARBs (*n*, %)	35 (39.8)	4 (26.7)	1 (6.3)	30 (52.6)	0.003
Antidiabetic treatment (*n*, %)					< 0.001
None	5 (5.7)	N/A	0 (0)	5 (8.8)	
Metformin	45 (51.1)	N/A	4 (25)	41 (71.9)	
SGLT2i	18 (20.5)	N/A	3 (18.8)	15 (26.3)	
GLP-1 analogues	13 (14.8)	N/A	1 (6.3)	12 (21.1)	
Insulin	31 (35.2)	N/A	15 (100)	16 (28.1)	
Hematocrit (%)	43.0 (3.1)	43.5 (4.5)	43.7 (2.4)	42.6 (2.9)	0.399
Glucose (mg/dL)	114.6 (38.9)	94.8 (9.4)	148.5 (67.6)	110.2 (25.4)	< 0.001
HbA1c (%)	6.4 (1.0)	5.3 (0.4)	7.0 (1.2)	6.5 (0.9)	< 0.001
LDL-C (mg/dL)	94.5 (35.0)	127.1 (32.5)	84.9 (24.9)	88.5 (33.60)	< 0.001
Triglycerides (mg/dL)	118.5 (96.3)	120.2 (46.3)	65.6 (30.4)	133.1 (112.7)	0.045
Creatinine (mg/dL)	0.83 (0.27)	0.79 (0.14)	0.79 (0.15)	0.86 (0.32)	0.525
eGFR (ml/min/1.73m^2^)	93.0 (25.1)	97.5 (23.4)	99.1 (21.1)	90.1 (26.4)	0.708
Urine creatinine (mg/dL)	193.8 (297.6)	435.4 (597.3)	110.1 (30.4)	159.6 (218.4)	0.135
Urine albumin (mg)	20.6 (68.3)	5.5 (2.8)	3.9 (2.5)	27.2 (80.8)	0.694
Urine albumin-to-creatinine ratio (mg/g)	118.5 (237.4)	27.5 (24.2)	39.2 (31.8)	157.2 (279.0)	0.641
hs-CRP (mg/L)	1.76 (1.74)	0.89 (0.70)	1.14 (0.79)	2.01 (1.94)	0.374

Abbreviations: *BMI* body mass index, *ACEi* angiotensin converting enzyme inhibitors, *ARBs* angiotensin receptor blockers, *SGLT2i* sodium-glucose transporter 2 inhibitors, *GLP-1* glucagon-like peptide 1, *HbA1c* hemoglobin A1c, *LDL-C* low-density lipoprotein cholesterol, *eGFR *estimated glomerular filtration rate, *hs-CRP* high sensitivity C-reactive protein

Data are presented as mean (SD) for continuous variables and *n* (%) for categorical variables

**Table 2:** Cardiac magnetic resonance parameters in healthy controls, type 1 and 2 diabetes patients

Parameter	Healthy controls (*n* = 12)	Type 1 Diabetes (*n* = 12)	Type 2 Diabetes (*n* = 47)	*P*-value
LV EDV (ml)	154.9 (42.3)	135.3 (30.7)	129.8 (31.0)	0.068
LV ESV (ml)	56.8 (18.1)	51.2 (13.3)	47.7 (14.7)	0.167
SV LV (ml)	88.2 (33.7)	84.1 (20.2)	79.4 (20.1)	0.442
Cardiac Index LV (L/min/m^2^)	**4.01 (0.67)**	**3.57 (0.68)**	**3.03 (0.46)**	**< 0.001**
SV RV (ml)	**98.3 (25.8)**	**85.1 (19.7)**	**80.3 (15.8)**	**0.013**
Cardiac Index RV (L/min/m^2^)	**4.01 (0.63)**	**3.61 (0.66)**	**2.96 (0.40)**	**< 0.001**
Min LA volume (ml)	27.9 (9.9)	26.5 (11.9)	30.4 (14.8)	0.686
Max LA volume (ml)	71.0 (17.7)	60.9 (18.6)	67.9 (19.5)	0.488
LA ejection fraction (%)	61.4 (8.6)	61.1 (6.2)	56.6 (11.5)	0.301
LA long axis strain (%)	**40.8 (21.3)**	**38.6 (17.8)**	**27.8 (11.7)**	**0.010**
RA long axis strain (%)	38.5 (10.4)	40.6 (18.6)	45.3 (39.2)	0.790
T1 native mapping (ms)	1,269.8 (34.5)	1,261.4 (73.4)	1,237.2 (124.8)	0.604
T2 mapping (ms)	49.3 (3.5)	49.6 (4.8)	50.8 (3.6)	0.411
ECV (%)	25.6 (3.3)	27.3 (3.6)	28.5 (6.3)	0.312
Global radial Strain (%)	35.0 (6.3)	32.4 (10.2)	35.1 (6.9)	0.616
Global radial Strain rate (%/ms)	2.17 (0.58)	2.18 (0.87)	2.02 (0.51)	0.689
Global radial displacement (degrees)	5.98 (0.92)	5.62 (1.90)	5.62 (0.79)	0.686
Global circumferential strain (%)	19.1 (2.3)	18.5 (3.5)	19.3 (2.2)	0.697
Global circumferential strain rate (%/ms)	1.14 (0.20)	1.23 (0.45)	1.09 (0.17)	0.329
Global circumferential displacement (degrees)	0.49 (0.75)	1.23 (1.12)	0.81 (0.79)	0.177
Global longitudinal strain (%)	16.6 (3.6)	17.0 (2.5)	17.2 (3.5)	0.875
Global longitudinal strain rate (%/ms)	1.22 (0.38)	1.19 (0.28)	1.02 (0.29)	0.114
Global longitudinal displacement (degrees)	2.5 (1.0)	3.3 (1.0)	3.2 (1.1)	0.183
Global longitudinal velocity (mm/s)	25.9 (12.2)	27.1 (10.3)	25.3 (9.3)	0.889
Resting perfusion Index basal region	0.17 (0.08)	0.21 (0.13)	0.21 (0.10)	0.643
Resting perfusion Index mid region	0.17 (0.06)	0.21 (0.14)	0.21 (0.11)	0.586
Resting perfusion Index apical region	0.20 (0.08)	0.24 (0.15)	0.24 (0.11)	0.608
Stress perfusion index basal region	0.16 (0.05)	0.15 (0.05)	0.22 (0.13)	0.157
Stress perfusion index mid region	0.16 (0.05)	0.16 (0.04)	0.21 (0.11)	0.224
Stress perfusion index apical region	0.17 (0.06)	0.17 (0.05)	0.21 (0.12)	0.344
Perfusion reserve index basal region	1.11 (0.55)	0.91 (0.34)	1.22 (0.82)	0.464
Perfusion reserve index mid region	1.01 (0.24)	0.94 (0.30)	1.06 (0.54)	0.712
Perfusion reserve index apical region	0.92 (0.22)	0.85 (0.29)	1.03 (0.70)	0.631
Perfusion index rest	0.18 (0.08)	0.22 (0.14)	0.22 (0.10)	0.528
Perfusion index stress	0.17 (0.05)	0.16 (0.04)	0.21 (0.11)	0.148
Signal intensity rest	1,400.9 (503.3)	1,437.3 (513.8)	1,442.5 (572.2)	0.980
Signal intensity stress	1,139.0 (310.4)	1,203.9 (469.8)	1,328.8 (574.4)	0.555

Abbreviations: *LV* left ventricular, *EDV* end-diastolic volume, *ESV* end-systolic volume, *SV* stroke volume, *RV* right ventricle, *LA* left atrium, *RA* right atrium, *ECV* extracellular volume

*P*-value is derived by one way analysis of variance (ANOVA)

Continuous variables are presented as mean (SD). Boldface values indicate statistical significance, which was set at the level of *P*-value < 0.05

**Table 3:** Echocardiographic parameters in healthy controls, type 1 and 2 diabetes patients

Parameter	Healthy controls (*n* = 13)	Type 1 Diabetes (*n* = 10)	Type 2 Diabetes (*n* = 38)	*P*-value
LVEF (%)	61.8 (7.0)	61.5 (6.5)	58.0 (6.6)	0.153
LV end-diastolic volume (ml)	94.4 (37.0)	73.8 (17.1)	76.8 (18.9)	0.071
LV end-systolic volume (ml)	37.3 (20.9)	29.0 (6.2)	32.5 (10.2)	0.340
LV internal diameter in diastole (mm)	49.2 (8.4)	44.2 (3.7)	46.3 (4.9)	0.107
LV internal diameter in systole (mm)	29.3 (8.5)	26.0 (3.9)	28.7 (5.5)	0.380
Interventricular septal thickness in diastole (mm)	**8.5 (1.1)**	**7.8 (1.5)**	**9.7 (1.6)**	**0.001**
Posterior wall thickness in diastole (mm)	8.3 (0.9)	8.0 (1.5)	9.0 (1.5)	0.091
Aortic valve annulus diameter (mm)	30.8 (5.3)	27.4 (1.3)	29.8 (4.1)	0.179
Ascending aorta diameter (mm)	**36.0 (5.2)**	**29.8 (2.6)**	**34.6 (4.5)**	**0.012**
LA diameter (mm)	**37.6 (5.6)**	**34.7 (4.7)**	**39.1 (4.4)**	**0.040**
LA volume index (ml/m^2^)	25.2 (6.1)	21.5 (8.3)	26.7 (8.4)	0.225
Peak atrial longitudinal strain – LA – Reservoir phase (%)	**27.6 (6.3)**	**38.8 (4.4)**	**28.7 (7.8)**	**0.018**
LA strain – Conduit phase (%)	**18.3 (7.9)**	**23.0 (6.3)**	**12.6 (4.3)**	**< 0.001**
Peak atrial contraction strain – LA – Contractile phase (%)	**-9.4 (5.5)**	**-16.0 (2.9)**	**-16.0 (6.2)**	**0.035**
RA volume index (ml/m^2^)	17.8 (5.7)	17.3 (3.7)	17.6 (6.9)	0.985
RA strain – Reservoir phase (%)	**34.1 (10.3)**	**41.2 (8.6)**	**31.0 (10.2)**	**0.036**
E/A Ratio	0.97 (0.23)	1.21 (0.43)	0.94 (0.29)	0.073
Deceleration time of E-wave (ms)	207.6 (43.7)	199.3 (56.0)	226.2 (65.8)	0.429
Early diastolic tissue Doppler velocity of septal mitral annulus (cm/s)	7.3 (2.4)	9.0 (2.0)	7.6 (1.6)	0.119
Early diastolic tissue Doppler velocity of lateral mitral annulus (cm/s)	**9.8 (3.7)**	**14.4 (3.2)**	**8.8 (2.2)**	**< 0.001**
E/E′ Ratio (Average of septal and lateral e′)	**6.9 (1.1)**	**6.0 (1.3)**	**8.9 (2.5)**	**0.002**
LV outflow tract diameter (mm)	20.9 (1.8)	19.0 (1.6)	20.3 (1.8)	0.059
LV cardiac output (L/min)	4.0 (1.2)	3.5 (0.8)	3.4 (1.1)	0.276
RVSTDI (S′ wave) (cm/s)	13.5 (2.9)	13.4 (2.4)	12.6 (2.5)	0.437
TAPSE (mm)	23.5 (4.7)	23.1 (3.2)	22.7 (4.0)	0.823
RVSP (mmHg)	20.0 (0.0)	21.1 (3.3)	21.2 (3.9)	0.743
LV GLS (%)	-19.9 (2.8)	-21.0 (1.7)	-19.3 (1.9)	0.088
RV GLS - Free Wall (%)	-27.2 (3.8)	-27.6 (3.6)	-25.4 (4.2)	0.307
PWV/GLS	**0.48 (0.14)**	**0.39 (0.06)**	**0.53 (0.12)**	**0.018**
TAPSE/RVSP	1.28 (0.29)	1.10 (0.19)	1.15 (0.33)	0.512
Global Work Index (mmHg%)	1,749.9 (164.4)	1,705.9 (172.9)	1,823.2 (288.6)	0.502
Global Constructive Work (mmHg%)	2,162.2 (265.3)	2,067.1 (253.4)	2,183.1 (348.1)	0.703
Global Wasted Work (mmHg%)	142.2 (68.9)	100.0 (41.6)	116.6 (59.0)	0.336
Global Work Efficiency (%)	92.8 (2.9)	94.7 (1.4)	93.9 (2.3)	0.241

Abbreviations: *LVEF* left ventricular ejection fraction, *LV* left ventricular, *LA* left atrium, *RA* right atrium, *RVSTDI* right ventricular systolic tissue Doppler imaging, *TAPSE* tricuspid annular plane systolic excursion,* RVSP* right ventricular systolic pressure, *GLS* global longitudinal strain, *PWV* pulse wave velocity

*P* value is derived by one way analysis of variance (ANOVA)

Continuous variables are presented as mean (SD). Boldface values indicate statistical significance, which was set at the level of *P*-value < 0.05

### Cardiac volumes, mass, and dimensions

PwD had significantly lower values of LV and RV cardiac index (3.14 vs. 4.01 L/min/m^2^ and 3.10 vs. 4.01 L/min/m^2^ respectively) as well as RV stroke volume (81.3 vs. 98.3 ml) compared with controls (*P* < 0.05 for all). Interventricular septal thickness was significantly increased in PwT2D (9.7 ± 1.6 mm) compared to PwT1D (7.8 ± 1.5 mm) and controls (8.5 ± 1.1 mm) (*P* = 0.001) (Table 3). LA diameter was also greater in T2DM (39.1 ± 4.4 mm) compared to T1DM (34.7 ± 4.7 mm) and controls (37.6 ± 5.6 mm) (*P* = 0.040). DM duration ≥ 10 years was associated with a mean decrease in global radial displacement of 1.15° (95% CI: -1.87° to -0.43°, *P* = 0.003) after multivariable adjustment. In contrast, after multivariable analysis, SGLT2i treatment did not present significant associations with markers of cardiac structure (Supplementary Table 1).

### Cardiac function

Left ventricular ejection fraction (LVEF) did not differ significantly among T2DM, T1DM, and control groups (*P* = 0.153), although PwT2D showed a trend toward lower LVEF. Diastolic function, as assessed by the E/E′ ratio, was significantly impaired in T2DM (8.9 ± 2.5) compared to controls (6.9 ± 1.1) and T1DM (6.0 ± 1.3) (*P* = 0.002). Associations of diabetes characteristics with structural and functional markers of cardiac function are illustrated in Fig. 2.

### Myocardial mechanics (Strain analysis)

Both PwT2D and PwT1D exhibited significantly reduced myocardial strain parameters compared with controls. GLS and global radial strain were lower in both DM groups, while LA strain was most impaired in PwT2D (27.8%) versus PwT1D (38.6%) and controls (40.8%) (*P* = 0.010). PwD exhibited significantly lower values of peak atrial contraction strain at contractile phase compared with controls (− 16.0% vs. − 9.4%, *P* = 0.009). Moreover, PwT2D also showed reduced LA conduit strain compared with PwT1D and controls (12.6% vs. 23.0% vs. 18.3% respectively, *P* < 0.001). DM duration > 10 years was negatively associated with GLS [β-coefficient=-2.06 (95% CI -4.08, -0.04), *P* = 0.046] and global radial strain [β-coefficient = − 7.50 (95% CI − 13.07, 1.92), *P* = 0.010] after adjustment for age, sex and hypertension. Tight control of DM was independently positively associated with global radial (β-coefficient = 0.82, *P* = 0.001) and global circumferential strain (β-coefficient = 0.32, *P* = 0.004).

### Perfusion and tissue characterization

After examining the association between DM and markers of perfusion at rest and stress, there were no significant differences in myocardial perfusion at rest or stress, nor in native T1 or T2 mapping values between PwD and controls (Table 2). DM duration was not associated with T1 or T2 alterations, but tight glycemic control was associated with a modest reduction in T2 mapping (mean decrease: 2.41 ms; 95% CI − 4.85 to − 0.01; *P* = 0.049). SGLT2i therapy did not significantly affect myocardial perfusion or tissue markers.

### Vascular function

#### Arterial stiffness

**Fig. 3:**
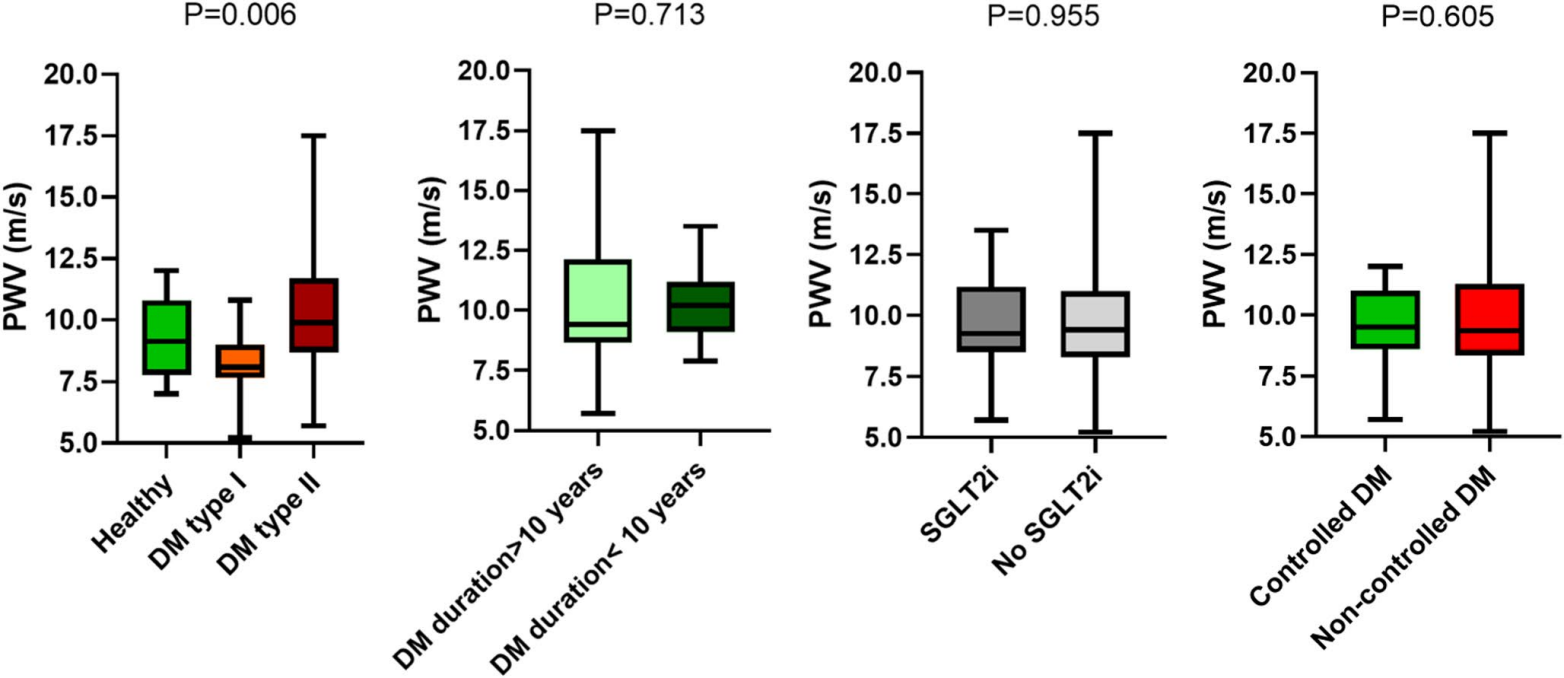
Associations of diabetes status according to type, duration and therapy with aortic stiffness. Abbreviations: *PWV* pulse wave velocity, *DM* diabetes mellitus, *SGLT2i* sodium-glucose transport protein 2 inhibitors

PwT2D had significantly increased PWV values compared with their counterparts with T1DM and controls (10.3 m/s vs. 9.3 m/s vs. 8.3 m/s respectively, *P* = 0.006) (Fig. 3). On the contrary, no differences in PWV values were observed according to DM duration, treatment with SGLT2i or tight control of DM (*P* > 0.05 for all) (Fig. 3). Subgroup analyses evaluating SGLT2i use were performed within PwD. Analyses of glucagon-like peptide-1 receptor agonist exposure were not performed due to limited statistical power and heterogeneity of treatment duration.

In patients who underwent CMR imaging, higher PWV values were associated with markers of diastolic dysfunction including increased diameter of LA (β-coefficient = 1.01, 95% CI 0.43–1.59) and LA volume index (β-coefficient = 2.03, 95% CI 0.90–3.16), (*P* < 0.05 for all). Furthermore, increased PWV was also positively correlated with diameter of interventricular septum (β-coefficient = 0.31, 95% CI 0.09–0.52), LV mass at the end of diastole (β-coefficient = 4237, 95% CI 1275–7198), as well as with the ratio of LV mass/ LV end-diastolic volume (β-coefficient = 19.43, 95% CI 0.68–38.17), even after adjustment for TRFs including age, sex and hypertension (*P* < 0.05 for all). Higher PWV was associated with impaired LV GLS after adjustment for the same core model (β-coefficient = 0.40, *P* = 0.038). In patients who underwent advanced echocardiography, PWV was positively associated with the global wasted work (GWW) (β-coefficient = 16.87, 95% CI 0.46–29.28), and inversely associated with global work efficiency (GWE) (β-coefficient = − 0.66, 95% CI − 1.14, − 0.18) (*P* < 0.05 for both), after adjustment for the same model.

### Arterial-ventricular coupling

In addition, PwT2D had significantly higher values of PWV/GLS ratio compared with PwT1D and controls (0.53 vs. 0.39 vs. 0.48 m/s% respectively, *P* = 0.025). No significant differences were observed according to DM duration. Tricuspid annular plane systolic excursion/ right ventricular systolic pressure (TAPSE/RVSP) ratio did not differ according to presence of DM. However, individuals with duration of DM ≥ 10 years had significantly lower values of TAPSE/RVSP ratio compared with those with DM duration < 10 years (1.04 ± 0.19 vs. 1.27 ± 0.40 respectively, *P* = 0.046) and DM duration ≥ 10 years was independently associated with a mean decrease of 0.270 (95% CI − 0.51, − 0.03, *P* = 0.027) in TAPSE/RVSP ratio after adjustment for age, sex and hypertension. On the contrary, markers of VAC did not differ according to tight control of DM or SGLT2i treatment.

### Diabetes and biomarkers

Regarding measurement of blood biomarkers, PwT2D had significantly higher levels of growth differentiation factor 15 (GDF-15) (1,702.3 ± 840.3 pg/mL vs. 666.9 ± 198.3 pg/mL, *P* = 0.027) compared with PwT1D (Supplementary Fig. 1). In PwD, GDF-15 levels were higher in patients receiving metformin compared with those not on metformin (1,846 ± 864 vs. 1,078 ± 303), although the difference did not reach statistical significance (*P* = 0.161). No other differences were observed in N-terminal pro-brain natriuretic peptide, interleukin-1b, interleukin-6 or ST2 according to type, duration, tight control of DM and SGLT2i treatment. Correlations of biomarkers with imaging parameters per DM status, DM duration, tight control of DM and SGLT2i treatment are presented in Table 4.

**Table 4:** Correlation of biomarkers with imaging parameters in (i) patients with DM, (ii) patients with DM duration ≥ 10 years, (iii) patients with tight control of DM and (iv) patients on SGLT2i treatment

Imaging parameters	EDV RV (CMR)	ESV LV (CMR)	LV cardiac output (echo)	RV cardiac output (echo)	IVSd (echo)	E/E′ Ratio (echo)	Peak atrial longitudinal strain (echo)	RA long axis strain (CMR)	LV GLS (echo)	RV GLS (echo)	Global longitudinal strain (CMR)	Global radial strain (CMR)	Global circumferential strain (CMR)	LV torsion (CMR)	Global longitudinal displacement (CMR)
i. DM presence	ii.	iii.	iv.	v.	vi.	vii.	viii.	ix.	x.	xi.	xii.	xiii.	xiv.	xv.	xvi.
Biomarker	**β-coefficients**														
NT-proBNP	− 0.01	− 0.01	**− 0.01** ^*^	**− 0.01** ^*^	0.01	**0.01** ^*^	**− 0.02** ^*^	0.00	0.00	0.00	0.00	0.00	0.00	0.00	0.00
IL-6	**− 7.50** ^*^	− 1.39	− 0.25	**− 0.30** ^*^	**0.48** ^*^	0.62	− 2.11	3.79	**0.69**	1.16	0.05	0.14	0.07	0.04	− 0.34
ST-2	1.56	0.69	0.05	0.06	-0.03	0.10	− 0.46	− 1.70	− 0.04	− 0.01	**− 0.20** ^*^	− 0.01	− 0.43	**− 0.04** ^*^	**− 0.08** ^*^
xvii. DM duration ≥ 10 years	xviii.	xix.	xx.	xxi.	xxii.	xxiii.	xxiv.	xxv.	xxvi.	xxvii.	xxviii.	xxix.	xxx.	xxxi.	xxxii.
NT-proBNP	− 0.01	− 0.01	0.00	0.00	0.00	0.00	− 0.01	0.00	0.00	0.00	0.00	0.00	0.00	0.00	0.00
IL-6	− 3.12	1.73	− 0.19	− 0.35	**0.55** ^*^	0.67	**− 4.16**	4.35	**0.75**	1.70	− 1.04	− 0.22	0.15	− 0.04	− 0.66
ST-2	0.33	− 0.03	0.08	**0.10** ^*^	− 0.01	0.06	0.12	− 1.18	0.00	0.02	− 0.25	− 0.06	0.00	**− 0.07** ^*^	− 0.11
xxxiii. Tight control of DM	xxxiv.	xxxv.	xxxvi.	xxxvii.	xxxviii.	xxxix.	xl.	xli.	xlii.	xliii.	xliv.	xlv.	xlvi.	xlvii.	xlviii.
NT-proBNP	− 0.03	**− 0.02** ^*^	**− 0.01** ^*^	**− 0.01** ^*^	0.00	0.00	− 0.01	0.03	0.00	0.00	0.00	0.00	0.00	0.00	0.00
IL-6	− 7.09	− 1.31	− 0.33	− 0.37	− 0.35	− 0.18	2.52	− 1.26	− 0.03	**4.23** ^*^	0.20	0.10	0.12	0.05	− 0.61
ST-2	− 1.05	− 0.39	0.07	0.08	− 0.01	0.06	0.37	1.46	− 0.05	− 0.07	**− 0.40** ^*^	**− 1.06** ^*^	− 0.01	**− 0.05** ^*^	**− 0.16** ^*^
xlix. SGLT2i treatment	l.	li.	lii.	liii.	liv.	lv.	lvi.	lvii.	lviii.	lix.	lx.	lxi.	lxii.	lxiii.	lxiv.
NT-proBNP	0.01	0.01	0.00	0.00	0.00	0.00	-0.02	-0.01	0.00	0.00	0.00	0.01	0.00	0.00	0.00
IL-6	− 13.58	− 4.24	− 0.25	− 0.28	0.11	0.17	− 1.06	0.27	0.99	**3.93** ^*^	0.65	1.26	**0.22** ^*^	− 0.08	0.19
ST-2	− 1.14	− 0.24	0.07	0.08	− 0.07	0.13	0.14	− 0.06	0.04	− 0.08	**− 0.37** ^*^	− 0.75	0.02	− 0.05	− 0.04

Abbreviations: *CMR* cardiac magnetic resonance, *echo* echocardiography, *EDV* end-diastolic volume, *RV* right ventricular, *ESV* end-systolic volume, *LV* left ventricular, *IVSd* interventricular septal thickness in diastole, *RA* right atrial, *GLS* global longitudinal strain; *NT-proBNP* N-terminal pro–B-type Natriuretic Peptide, *IL-6* interleukin-6, *DM* diabetes mellitus, *SGLT2i* sodium–glucose cotransporter 2 inhibitor

Boldface values and asterisk indicate statistical significant associations. Statistical significance was set at the level of p-value < 0.05

### Associations between LFNIT and GDF-15 with indices of cardiometabolic function in patients with DM

Following CMR imaging, elevated non-alcoholic fatty liver disease score (NFS) was associated with markers of diastolic dysfunction, including increased diameter (β-coefficient = 2.23, *P* = 0.006) and maximum volume of LA (β-coefficient = 4.85, *P* = 0.042). Moreover, elevated NFS was associated with increased LV mass (β-coefficient = 6,195, *P* = 0.022) and reduced LVEF (β-coefficient = − 3.12, *P* = 0.017). Both NFS and Fibrosis-4 (FIB-4) were associated with reduced RV cardiac index (β-coefficient= − 0.13 and β-coefficient= − 0.40 respectively, *P* < 0.05 for both). FIB-4, but not NFS, was positively associated with GWW and inversely associated with GWE (*P* < 0.05 for both). After adjustment for age, sex and type of diabetes, GDF-15 was positively associated with LA volume index (β-coefficient = 0.009, *P* = 0.033) and LV internal diameter at the end of systole (β-coefficient = 0.008, *P* < 0.001) but inversely associated with peak atrial longitudinal strain of LA at reservoir phase (β-coefficient = − 0.007, *P* = 0.009) and LVEF (β-coefficient= − 0.005, *P* = 0.029).

## Discussion

Our study provides the first integrated evaluation of imaging, clinical, and biochemical markers of subclinical myocardial compromise associated with DCM. Using both echocardiography and CMR, we show that DM status, duration and glycemic control were differentially associated with early myocardial changes of DCM. Importantly, in patients without HF, these findings highlight distinct pathways and offer novel insights into the earliest stages of disease progression.

In our study, both T1DM and T2DM were associated with reduced cardiac index and stroke volume, while PwT2D showed greater impairments in LA strain and diastolic function. Moreover, longer DM duration was associated with worsening myocardial deformation, including lower GLS, radial strain, and displacement. Tight glycemic control was modestly associated with improved strain parameters. In contrast, myocardial tissue characterization and perfusion indices did not differ significantly by DM status or duration, suggesting early functional rather than structural changes in DCM.

### Left ventricular and atrial function and myocardial tissue composition

PwD exhibited significant reductions in LV and RV cardiac indices compared to controls. As no differences were observed in LVEF, this reduction in cardiac indices may reflect impaired cardiac filling [35]. In line with this, PwT2D showed reduced LA strain, suggesting early diastolic dysfunction. DCM, particularly in T2DM, often first presents as isolated diastolic dysfunction driven by increased myocardial stiffness, ultimately leading to symptoms of HFpEF [36]. Given that traditional measures of LA size and function did not differ by DM status, strain-based parameters may serve as more sensitive biomarkers for identifying early atrial involvement.

No alterations in myocardial tissue characterization in terms of T1, T2 mapping and ECV were noted in PwD. T1 mapping and ECV are established CMR-based markers of myocardial fibrosis, which is a hallmark of DCM, where a profibrotic response leading to extracellular matrix remodeling is induced by mitochondrial dysfunction, oxidative stress and inflammation [36]. Our findings align with the lack of difference in GDF-15 levels across DM status and duration, further supporting the absence of detectable fibrosis in this cohort. Although a recent meta-analysis demonstrated slightly higher ECV values in PwD as compared to controls, with no detectable differences in T1 mapping 37, these differences were minimal and may reflect variability across study populations and imaging protocols. Collectively, our results suggest that in well-characterized diabetic individuals without overt CV disease, functional myocardial impairment may precede detectable structural remodeling, although causality cannot be inferred from this cross-sectional study.

Whereas myocardial microvascular dysfunction is typical of DCM^38^, in our cohort no differences were observed in terms of semi-quantitative parameters of rest and stress myocardial perfusion. To this regard, Li et al. [39] observed reduced global myocardial perfusion reserve index in PwT2D with overt HFpEF, as opposed to PwT2D without HF. Our analysis included PwD without HF and other common causes of cardiomyopathy and we hypothesize that myocardial perfusion impairment develops and worsens as the disease progresses, but this cannot be confirmed with the current study design. However, these findings should be interpreted with caution. Myocardial perfusion was assessed using semi-quantitative indices, which, although widely used, may be less sensitive than fully quantitative myocardial blood flow measurements for detecting early microvascular dysfunction. In addition, perfusion analyses were performed in a relatively small subgroup of participants, and the study population consisted of asymptomatic individuals without HF, who likely represent an early stage of disease. Therefore, the absence of significant differences may reflect limited statistical power and potential type II error rather than true absence of microvascular impairment. Larger studies using fully quantitative perfusion techniques are needed to better define the presence and timing of microvascular dysfunction in early DCM.

Our analysis indicates that prolonged DM duration was associated with deteriorations in myocardial mechanics, as evidenced by decreased GLS and radial displacement. Conversely, tight glycemic control was positively correlated with higher global radial and circumferential strains, although it was inversely associated with LV torsion. While causality cannot be determined, these findings suggest that while stringent glycemic management may mitigate certain aspects of myocardial dysfunction, it may also have complex effects on myocardial mechanics.

#### Right ventricular function

The TAPSE/RVSP ratio is a novel non-invasive marker of efficiency of the right heart-pulmonary circulation system, whose role in the context of DCM without concomitant HF has never been studied before. It carries a significant prognostic value in different clinical settings such as tricuspid regurgitation, HF and arterial pulmonary hypertension [40, 41]. The TAPSE/RVSP ratio, was significantly decreased in individuals with DM duration ≥ 10 years. A longer duration of DM was also associated with alterations in RV function, expressed in terms of RV-pulmonary artery coupling. Accordingly, in a cohort of patients with HF, worse RV-pulmonary artery coupling and LV filling pressures were observed in PwD, as compared to euglycemic HF patients [42]. Collectively, these findings suggest that the metabolic derangements of DM may affect both ventricles, but causal relationships cannot be established.

### Arterial stiffness, coupling and LV mechanics in patients with diabetes

In terms of vascular function, PwT2D exhibited elevated arterial stiffness, as reflected by higher PWV, that has consistently been shown to predict CV and all-cause mortality independently of TRFs in diabetes [43]. In our study, elevated PWV correlated with larger LA dimensions, increased septal thickness, higher LV mass, and an increased LV mass-to-volume ratio, suggesting concentric remodeling and diastolic dysfunction in the early stages of DCM. These associations underscore the possible detrimental hemodynamic effects of arterial stiffening -mediated by aging, TRFs, inflammation, and oxidative stress- on LV performance [44]. Furthermore, higher PWV was associated with less negative GLS values, as well as impaired myocardial efficiency, demonstrated by higher GWW and lower GWE, reflecting the excess energy required to maintain cardiac output under increased afterload. Importantly, PwT2D showed a higher PWV/GLS ratio compared with both PwT1D and controls, indicating altered arterial–ventricular interactions rather than proving a causal effect. The physiological rationale for using the PWV/GLS ratio as a surrogate of VAC lies in the integration of arterial load and myocardial deformation into a single non-invasive metric [45, 46]. Arterial stiffness, as assessed by PWV, is a major determinant of pulsatile afterload and influences myocardial wall stress and energy demand [47]. In parallel, GLS reflects longitudinal myocardial fiber shortening and is a sensitive marker of subclinical systolic dysfunction, particularly in early DCM [48]. The PWV/GLS ratio therefore provides an integrative estimate of the interaction between arterial stiffness and myocardial contractile performance, which may be particularly relevant in cardiometabolic disease states where vascular and myocardial abnormalities develop in parallel [49]. Collectively, our findings further support the clinical value of validated non-invasive surrogate imaging endpoints for the early identification of CV dysfunction in asymptomatic populations at increased cardiometabolic risk. By focusing on GLS as the primary cardiac endpoint and PWV as the primary peripheral vascular parameter, our study reinforces their role as early markers of subclinical myocardial andvascular dysfunction that may precede overt CVD.

#### Liver fibrosis and biomarkers

LFNITs scoring systems (NFS and FIB-4) were associated with multiple cardiac structural and functional parameters, particularly diastolic dysfunction and LV remodeling.This is in line with prior literature linking liver fibrosis scores to diastolic impairment and suggesting a potential mechanistic link between hepatic fibrosis and myocardial dysfunction, especially in PwD, possibly mediated through shared inflammatory and fibrotic pathways [50–53]. Moreover, among individuals who underwent advanced echocardiographic assessment, FIB-4 was correlated with subclinical myocardial inefficiency at myocardial work analysis. Among the other biomarkers studied, only GDF-15 was associated with variables reflecting adverse cardiac remodeling, in line with its known role in cardiometabolic signaling in the context of DM [54].

#### Limitations

Limitations of the present study are its cross-sectional nature, with inherents bias, and its single-center nature, which may require further multicenter prospective cohort studies to validate our finding; however it ensures consistency and standardization. Moreover, the population of our study probably represents an early and subclinical phase of DCM, as the patients included were not evaluated primarily for HF symptoms. As a result, we did not capture some of the abnormalities that have been described in more advanced stages of DCM, but we are able to inform on the early changes that may be observed after the diagnosis of DM is made. Future longitudinal analysis of the participants will better inform how our current findings translate to DCM progression and risk. Furthermore, the limited sample size should be acknowledged, albeit it is counterbalanced by the comprehensiveness of imaging and biochemical evaluation. Multiple regression analyses were performed across several imaging, biomarker, and metabolic parameters without formal correction for multiple testing, increasing the risk of type I error [55]. Although co-primary outcomes were prespecified and powered, all other associations observed for secondary imaging, biomarker, and liver fibrosis parameters should be interpreted as hypothesis-generating and require validation in adequately powered prospective cohorts. In addition, although multivariable models were restricted to a limited number of clinically relevant covariates, the relatively small and unbalanced subgroup sizes may still increase the risk of model overfitting and limit the stability of adjusted estimates. Furthermore, CMR tissue characterization and perfusion analyses were conducted in smaller subgroups and were not primary study endpoints. Therefore, the absence of significant differences in T1, T2, ECV, and perfusion parameters may reflect limited statistical power and potential type II error, particularly for detecting subtle structural or microvascular alterations in this asymptomatic population. The PWV/GLS ratio is an indirect surrogate and does not replace gold-standard VAC assessment derived from pressure–volume loop analysis, such as the ratio of arterial elastance to ventricular elastance [56]. In addition, both PWV and GLS are influenced by loading conditions, blood pressure, heart rate, and measurement variability, which may affect the stability of the ratio [57, 58]. These limitations may be particularly relevant in asymptomatic diabetic populations, where hemodynamic abnormalities are subtle and dynamic, and small variations in either vascular or myocardial measurements may disproportionately influence the calculated ratio [9, 59]. Therefore, the PWV/GLS ratio should be interpreted as an exploratory and hypothesis-generating marker rather than a definitive measure of VAC. Finally, although liver fibrosis scores were evaluated, quantitative liver MRI could have provided a more precise assessment of hepatic steatosis and fibrosis burden. Future multimodal imaging studies integrating liver MRI with cardiac imaging may further clarify heart–liver metabolic interactions in diabetes. However, the aim of this study was to explore the association of LFNITs and imaging-defined markers of DCM using advanced CV imaging, irrespectively of formal NAFLD diagnosis.

## Conclusions

In conclusion, this comprehensive imaging-based study highlights the early and multifaceted impact of DM on cardiac structure and function, before the onset of clinically overt HF. Advanced imaging revealed subclinical myocardial dysfunction, particularly diastolic impairment, altered myocardial mechanics and inefficient interaction between the heart and vasculature. Liver fibrosis scores were associated with cardiac remodeling, underscoring systemic metabolic involvement. Overall, these findings, while not implying causality, emphasize the importance of early and integrated CV assessment in PwD, provide insights into metabolic pathways of DCM and identify potential early markers for further investigation.

## Supplementary Information

Below is the link to the electronic supplementary material.

Supplementary Material 1
