# Supplementary material for: Associations between cardiometabolic traits and diabetic cardiomyopathy: an imaging-based analysis
Source: Acta Diabetol. 2026 Apr 15;63(6):1067–81. doi: 10.1007/s00592-026-02687-4 (PMC13272257; doi:10.1007/s00592-026-02687-4)
Supplement: Supplementary file 1 — Supplementary Material 1 [file 592_2026_2687_MOESM1_ESM.docx]

**Supplementary Material**

**Supplementary Methods**

**Sample size calculation**

Sample size estimation was performed a priori based on the co-primary study endpoints, global longitudinal strain (GLS) and pulse wave velocity (PWV), as markers of early myocardial and vascular dysfunction, respectively.

The calculation was informed by previously published studies evaluating myocardial strain and arterial stiffness in people living with diabetes using advanced cardiovascular imaging modalities.^1, 2^ Expected effect sizes were derived from reported differences in GLS and PWV between diabetic and non-diabetic populations. Assuming a two-sided alpha level of 0.05 and a statistical power of 80%, the estimated sample size was sufficient to detect clinically meaningful differences in the co-primary endpoints.

The calculation was informed by previously published studies evaluating myocardial strain and arterial stiffness in people living with diabetes using advanced cardiovascular imaging modalities. Expected effect sizes were derived from reported differences in GLS and PWV between diabetic and non-diabetic populations.

Assuming:

- Two-sided alpha level of **0.05**
- Statistical power of **80%**
- Expected between-group difference of **2% in GLS**
- Expected between-group difference of **1.0 m/s in PWV**
- Estimated standard deviation of **≈2.5% for GLS**
- Estimated standard deviation of **≈1.2m/s for PWV**

the estimated sample size was sufficient to detect clinically meaningful differences in the co-primary endpoints.

Given the advanced imaging phenotyping and mechanistic focus of the study, the final sample size was considered appropriate to detect early subclinical cardiovascular alterations and to explore associations between cardiometabolic traits and imaging biomarkers.

*Echocardiography*

The echocardiographic images were analyzed using a commercially available 2D speckle tracking software package (EchoPAC PC version 204; GE Medical Systems, Milwaukee, WI, USA). Speckle tracking parameters were derived for the left ventricle (LV), right ventricle (RV) and left atrium (LA). Standard apical four-chamber, three-chamber, and two-chamber views were acquired with frame rates ranging from 60 to 100 frames per second. For each view, three cardiac cycles were recorded to facilitate optimal cycle selection during offline analysis.

From the three apical views, GLS, rotation, and circumferential strain of the LV were assessed. Additionally, myocardial global work index (GWI) -defined as the area under the pressure-strain loop from mitral valve closure to mitral valve opening (mm Hg%)- was calculated. Global constructive work (GCW), reflecting the sum of positive work during systolic shortening and negative work during isovolumetric relaxation (mm Hg%), as well as global wasted work (GWW), representing energy loss due to negative work during systole and positive work during isovolumetric relaxation (mm Hg%), were also computed. Myocardial global work efficiency (GWE, %) was determined as the ratio of constructive work to the sum of constructive and wasted work.^3^

RV longitudinal strain was measured in a blinded manner from four-chamber view images. For atrial strain assessment, longitudinal strain and strain rate curves were generated for six atrial segments using apical four- and two-chamber views. LA systolic strain was quantified at the end of the reservoir phase.

*Cardiac Magnetic Resonance Imaging*

LV myocardial strain was assessed using a 3-dimensional tissue tracking module, which involved loading long-axis two-chamber, four-chamber, and short-axis cine images. Through feature-tracking analysis, key parameters such as strain, strain rate and displacement in radial, circumferential, and longitudinal directions were automatically calculated. LA and right atrium strain was automatically derived after manually tracing in the apical four-chamber and two-chamber views the endocardial and epicardial border contours.

Stress myocardial perfusion was executed after intravenous adenosine infusion at a dose of 140 μg/kg/min for 4 minutes. For semi-quantitative perfusion analysis, endocardial and epicardial contours were manually outlined, and a region of interest was selected within the LV blood pool. Myocardial perfusion upslopes (perfusion index) during rest and stress were computed using a five-point linear fit model of signal intensity versus time, normalized to the upslope of the LV blood pool. The myocardial perfusion reserve index was defined as the ratio of stress to rest perfusion upslopes, each normalized to the corresponding LV blood pool upslope, thereby reflecting the vasodilatory capacity of the coronary microcirculation. The LV myocardium was segmented into 16 regions according to the American Heart Association guidelines, and perfusion parameters were calculated automatically.

*Ventricular-arterial coupling*

Ventricular-arterial coupling (VAC) reflects the dynamic relationship between the heart’s contractile function and the arterial system, playing a crucial role in determining CV efficiency and overall performance.^4^ VAC can be evaluated by non-invasive markers. The **pulse wave velocity to global longitudinal strain (PWV/GLS)** ratio integrates arterial stiffness and myocardial deformation, providing insight into LV-arterial interaction.^5^ Similarly, the **tricuspid annular plane systolic excursion to right ventricular systolic pressure (TAPSE/RVSP)** ratio serves as a surrogate of RV-arterial coupling, especially relevant in pulmonary hypertension and RV dysfunction.^6^ Together, these markers offer a more comprehensive, chamber-specific view of VAC and its clinical implications.

*Metabolic profile, liver fibrosis and biomarkers measurement*

All participants underwent comprehensive medical documentation, including history, clinical, and laboratory exams to assess CV risk factors. Tight control of DM was defined as an hemoglobin A1c of 6.5% or lower. At baseline, patients underwent blood sampling for metabolic profiling and blood-based LFNITs—Fibrosis-4 index (FIB-4) and non-alcoholic fatty liver disease (NAFLD) fibrosis score (NFS)—were retrospectively calculated for all participants with available parameters at baseline. Plasma levels of growth differentiation factor-15 (GDF-15), interleukin-1β (IL-1β), interleukin-6 (IL-6), N-terminal pro-brain natriuretic peptide (NT-proBNP), and ST2 were measured using commercially available enzyme-linked immunosorbent assay (ELISA) kits or multiplex immunoassay platforms, following the manufacturers' instructions.

*Statistical analysis*

Because not all participants underwent both echocardiography and CMR imaging, the effective sample size differed across analyses depending on data availability for each outcome. Models including echocardiographic outcomes were based on participants with complete echocardiographic data (n=62), while models including CMR-derived parameters were restricted to those with available CMR measurements (n=77).

**Supplementary Table 1. Main echocardiographic and CMR parameters according to SGLT2i treatment**

|  | **SGLT2i (n=18)** | **No SGLT2i (n=70)** | **P-value** |
| --- | --- | --- | --- |
| **LVEF (%) (echo)** | 59.8 (6.2) | 58.8 (6.7) | 0.685 |
| **Cardiac Index LV (L/min/m^2^) (CMR)** | 2.96 (0.55) | 3.21 (0.56) | 0.164 |
| **LV cardiac output (L/min) (CMR)** | 5.8 (1.2) | 6.4 (1.2) | 0.122 |
| **Cardiac Index RV (L/min/m^2^) (CMR)** | 2.92 (0.56) | 3.16 (0.54) | 0.190 |
| **RV cardiac output (L/min) (CMR)** | 5.8 (1.3) | 6.3 (1.1) | 0.136 |
| **T1 native mapping (ms) (CMR)** | 1223,1 (116.8) | 1,245.9 (117.4) | 0.553 |
| **T2 mapping (ms) (CMR)** | 51.2 (5.0) | 50.3 (3.6) | 0.496 |
| **LV GLS (%) (CMR)** | -19.8 (1.9) | -19.7 (2.0) | 0.868 |
| **RV GLS - Free Wall (%) (CMR)** | -25.6 (4.3) | -26.2 (4.2) | 0.746 |
| **PWV/GLS** | 0.46 (0.14) | 0.51 (0.13) | 0.373 |
| **TAPSE/RVSP** | 1.25 (0.28) | 1.14 (0.31) | 0.482 |
| **Global Work Index (mmHg%) (echo)** | 1,807.0 (345.5) | 1,805.0 (224.8) | 0.987 |
| **Global Constructive Work (mmHg%) (echo)** | 2,202.0 (457.4) | 2,158.0 (249.9) | 0.764 |
| **Global Wasted Work (mmHg%) (echo)** | 115.7 (55.9) | 113.8 (55.2) | 0.938 |
| **Global Work Efficiency (%) (echo)** | 93.9 (2.1) | 94.2 (2.1) | 0.739 |
| Abbreviations: SGLT2i, sodium-glucose cotransporter 2 inhibitors; LVEF, left ventricular ejection fraction; LV, left ventricular; RV, right ventricular; GLS, global longitudinal strain; PWV, pulse wave velocity; TAPSE, tricuspid annular plane systolic excursion; RVSP, right ventricular systolic pressure.  Subgroup analyses evaluating SGLT2 inhibitor use were performed within PwD. | | | |

**Supplementary Figure 1. GDF-15 values according to type of diabetes.**

Abbreviations: DM, diabetes mellitus; GDF-15, growth differentiation factor-15.

**References**

1. Tadic M, Ilic S, Cuspidi C, Kocijancic V, Celic V. Prediabetes, diabetes and left heart deformation. *Rev Esp Cardiol (Engl Ed)*. Dec 2014;67(12):1062-4. doi:10.1016/j.rec.2014.07.019

2. Tanaka K, Kawai T, Saisho Y, et al. Relationship between Stage of Diabetic Retinopathy and Pulse Wave Velocity in Japanese Patients with Type 2 Diabetes. *J Diabetes Res*. 2013;2013:193514. doi:10.1155/2013/193514

3. Briasoulis A, Bampatsias D, Petropoulos I, et al. Left ventricular myocardial work improves in response to treatment and is associated with survival among patients with light chain cardiac amyloidosis. *Eur Heart J Cardiovasc Imaging*. Apr 30 2024;25(5):698-707. doi:10.1093/ehjci/jead351

4. Chantler PD, Lakatta EG, Najjar SS. Arterial-ventricular coupling: mechanistic insights into cardiovascular performance at rest and during exercise. *J Appl Physiol (1985)*. Oct 2008;105(4):1342-51. doi:10.1152/japplphysiol.90600.2008

5. Holm H, Magnusson M, Jujić A, Bozec E, Girerd N. How to calculate ventricular-arterial coupling? *Eur J Heart Fail*. Apr 2022;24(4):600-602. doi:10.1002/ejhf.2456

6. Tello K, Dalmer A, Axmann J, et al. Reserve of Right Ventricular-Arterial Coupling in the Setting of Chronic Overload. *Circ Heart Fail*. Jan 2019;12(1):e005512. doi:10.1161/circheartfailure.118.005512
